# Vitamin D metabolism in thyrotoxicosis. Therapeutic aspects derived from an old observation

**DOI:** 10.1111/j.1742-1241.2009.02033.x

**Published:** 2009-08

**Authors:** C Veletzas

**Affiliations:** Illission 7 Athens, Endocrine Unit, University of AthensGreece E-mail: impression713@yahoo.gr

To the Editor:

In an article of 2007 about vitamin D deficiency, it was reported that low 25OH-vitD (25hydroxyvitaminD) plasma levels are found in thyrotoxicosis, with thyrotoxicosis being the last in the list of acquired causes of vitamin D deficiency (1). We first demonstrated low 25OH-vitD plasma levels in thyrotoxicosis (2), as well as the fact that the transformation of vitamin D into 25OH-vitD is 2.5 to eight times faster in thyrotoxic subjects in comparison to healthy controls (3).

**Figure 1:**
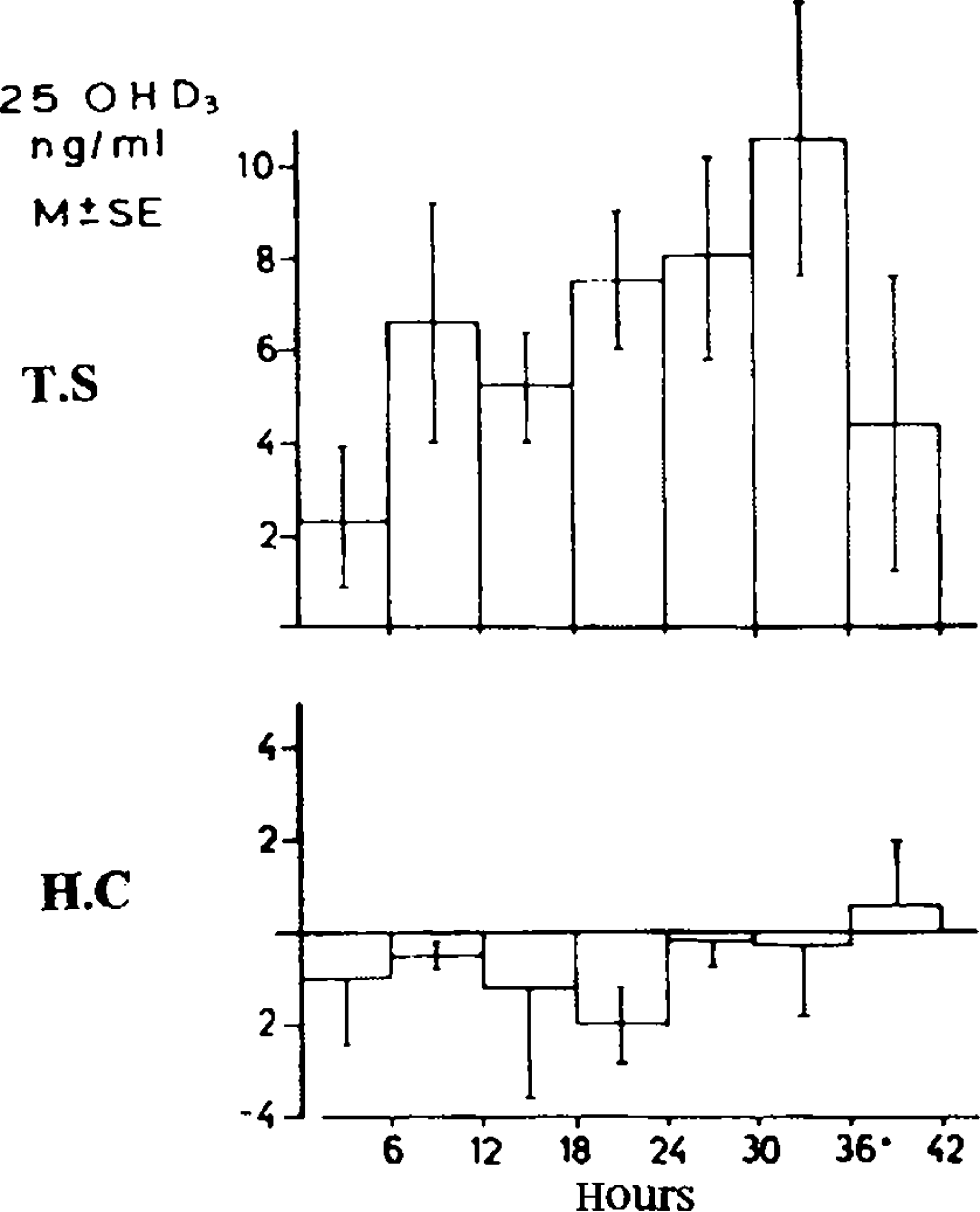
25hydroxyvitaminD plasma levels estimated the same period in three thyrotoxic subjects (TS) and three healthy controls (HC), all males matched for age and body weight after i.v. injection of 1 mg of vitamin D

In an unpublished report, the acceleration of hydroxylation of vitamin D into 25OH-vitD in thyrotoxic subjects (HC) was depicted against that of healthy controls (HC) (Figure 1).

When our observations were made, the lower normal limits of 25OH-vitD plasma levels were ill defined as various cut points were used for a long time to define vitamin D inadequacy fluctuating from 9 ng/ml to 30 ng/ml (4). Therefore, it was inconvenient to consider hypovitaminosis D with 25OH-vitD plasma levels between 9 ng/ml and 30 ng/ml.

These nebulous data about the lower normal limits of 25OH-vitD plasma levels resulted in the lack of concern regarding the acceleration of vitamin D metabolism in thyrotoxicosis, although the osteomalacic component has been mentioned long ago in thyrotoxic bone disease (5).

In recent years, a level of 30 ng/ml or greater of 25OH-vitD plasma levels was approved definitely as the lower normal level in humans, while the toxic levels were defined as greater than 150 ng/ml (1). The new data validated the ascertained acceleration of vitamin metabolism in thyrotoxicosis as thyrotoxic subjects are at risk for drop of 25OH-vitD plasma levels below the level of 30 ng/ml. In addition, hyperthyroidism is an endocrinopathy, which may run for a long time without the patient seeking medical intervention. Hence, the vitamin D depot in the body may decline in the levels of inadequacy or overt deficiency with the all known consequences. Especially prone to this risk are the thyrotoxic subjects of old age or institutionalised as well as those confined at home. A similar condition may be observed in patients taking suppressive doses of thyroxine or incorrectly increased doses of replacement therapy.

The aim of this issue is to establish in all patients who are under the influence of increased thyroid hormones, a testing of their vitamin D status parallel to the testing of their thyroid status, as well as the follow-up of their 25OH-vitD plasma levels during the medical supervision of the patient.

The intervention with vitamin D when insufficiency or deficiency of this vitamin ensues will avert the deleterious effect of this inadequacy especially to the bones.

This substance ‘vitamin D-hormone’, on the basis of the above mentioned, must be considered as a serious agent in the restoration of health after the elapse of toxicosis in thyrotoxic patients, given the fact that many are unaware of this kind of inadequacy in thyrotoxic subjects.
